# SEQUENCE ANALYSIS OF CARDIOMETABOLIC MULTIMORBIDITY AND ASSOCIATION WITH SUBSEQUENT DEMENTIA

**DOI:** 10.1093/geroni/igad104.0758

**Published:** 2023-12-21

**Authors:** Corey Nagel, Heather Allore, Anda Botoseneanu, Jeffrey Kaye, Jason Newsom, Nicholas Bishop, Ana Quiñones

**Affiliations:** University of Arkansas for Medical Sciences, Little Rock, Arkansas, United States; Yale University, New Haven, Connecticut, United States; University of Michigan, Ann Arbor, Michigan, United States; Oregon Health & Science University, Portland, Oregon, United States; Portland State University, Portland, Oregon, United States; University of Arizona, Tucson, Arizona, United States; Oregon Health & Science University, Portland, Oregon, United States

## Abstract

Sequence analysis is used in the social sciences to examine patterns of events occurring across the life course, but there are few examples of its use in multimorbidity research among older adults. We used sequence analysis to identify longitudinal patterns of cardiometabolic multimorbidity over a five-year period among participants in the National Health and Aging Trends Study (N=5,218). Multimorbidity sequences were constructed using self-reported diagnosis of diabetes, heart disease, stroke, and myocardial infarction (MI) assessed annually. Death was included as an absorbing state, yielding a total of 281 distinct sequences. We calculated sequence dissimilarity using optimal matching then used hierarchical clustering to identify seven distinct sequence clusters. The largest cluster (46.2%) was characterized by no baseline cardiometabolic disease and minimal incident disease across the 5-year period. Three clusters were characterized by stable sequences: diabetes (13.1%), heart disease (7.5%), and MI or stroke (7.3%) across the 5-year period. Two clusters exhibited a high rate of incident cardiometabolic disease during the 5-year period, one among persons with no baseline disease (9.6%) and one with rapid accumulation of cardiometabolic multimorbidity (5.3%). Finally, one cluster largely contained persons who died during the study period (11.0%). Compared to those with no baseline and minimal incident cardiometabolic disease, the odds of subsequent dementia were significantly higher among the cluster without prior disease who developed incident cardiometabolic disease (OR= 1.61, 95% CI:1.07,2.43) and the cluster with high cardiometabolic multimorbidity (OR=2.77, 95% CI:1.84,4.18). These findings contribute to our understanding of the impact of cardiometabolic multimorbidity on cognitive health.

